# Effect of Alcohol Administration on Mg^2+^ Homeostasis in H9C2 Cells

**DOI:** 10.4172/2329-9517.1000179

**Published:** 2014-10-23

**Authors:** Huy Nguyen, Andrea Romani

**Affiliations:** Department of Physiology and Biophysics, School of Medicine, Case Western Reserve University, Cleveland, OH 44106, USA

**Keywords:** H9C2 cells, Magnesium, Cardiac, Ethanol, CyP4502E1

## Abstract

Alcoholic cardiomyopathy represents one of the main clinical complications in chronic alcoholics. This pathology contrasts the seemingly beneficial effect of small doses of alcohol on the cardiovascular system. Studies carried out in liver cells exposed acutely or chronically to varying doses of EtOH indicate that intrahepatic alcohol metabolism results in a major loss of cellular Mg^2+^. To investigate whether EtOH administration also induced Mg^2+^ extrusion in cardiac cells, H9C2 cells were exposed to varying doses of EtOH for short- or ling-term periods of time. The results indicate that H9C2 cells exposed to EtOH doses higher than 0.1% (v/v, or 15 mM) extruded Mg^2+^ into the extracellular medium on a time- and dose-dependent manner. Consistent with the involvement of cyP4502E1 in metabolizing EtOH, administration of chloro-methiazole (CMZ) as an inhibitor of the cytochrome prevented EtOH-induced Mg^2+^ loss to a large extent. EtOH-induced Mg^2+^ extrusion was also prevented by the administration of di-thio-treitol (DTT) and n-acetyl-cysteine (NAC), two agents that prevent the negative effects of ROS formation and free radicals generation associated with EtOH metabolism by cyP4502E1.

Taken together, our data indicate that Mg^2+^ extrusion also occur in cardiac cells exposed to EtOH as a result of alcohol metabolism by cyP4502E1 and associated free radical formation. Interestingly, Mg^2+^ extrusion only occurs at doses of EtOH higher than 0.1% administered for an extended period of time. The significance of Mg^2+^ extrusion for the onset of alcoholic cardiomyopathy remains to be elucidated.

## Introduction

Magnesium (Mg^2+^), the second most abundant cation within mammalian cells after potassium [1], is highly represented within nucleus, mitochondria, and endo-sarco-plasmic reticulum [1–3]. Total Mg^2+^ concentrations ranging between 16–20 mM have been measured within each of these compartments [1–3]. As for the cytoplasm, approximately 4–5 mM Mg^2+^ is present in the form of a complex with ATP, phosphocreatine and other phospho-nucleotides [2,4], leaving the free Mg^2+^ concentration ([Mg^2+^]i) to range between 0.5 and 1 mM [2,3]

A similar distribution has been measured in the majority of mammalian cells including cardiac myocytes [2,3], and in the absence of hormonal or metabolic stimuli, no major changes in cellular Mg^2+^ concentration are detected. Following stimulation of adrenergic receptors by catecholamine or isoproterenol [5–7] a major and rapid extrusion of Mg^2+^ across the myocyte cell membrane is observed [2,3], with minimal or no changes in [Mg^2+^]_i_ [8]. The mechanism responsible for Mg^2+^ extrusion across the sarcolemma of cardiac cells has been identified with a cAMP-activated Na^+^/Mg^2+^ exchanger both in cardiac myocytes [5–7] and sarcolemmal vesicles [9].

Alcohol consumption as one of the main causes of Mg^2+^ loss from alcohol-responsive tissues including liver [10]. Our laboratory has reported that acute ethanol (EtOH)^1^ administration to liver cells causes two well distinct effects. By inhibiting anaerobic glycolysis in a dose-dependent manner, EtOH transient decreases cellular ATP [11], reducing its ability to complex cytoplasmic Mg^2+^. As cytosolic [Mg^2+^] i increases, Mg^2+^ is extruded through the Na^+^/Mg^2+^ exchanger [11]. In addition, EtOH inhibits Mg^2+^ entry into the cell for at least 60 min after alcohol removal [12], delaying the restoration of proper cellular Mg^2+^ homeostasis. Similar effects occur on a more prolonged time period in liver cells of animals chronically exposed to alcohol in the diet [12,13].

Cardiac myocytes are also negatively affected by EtOH administration, and alcoholic cardio-myopathy constitutes one of the major pathological complications in alcoholics [14,15]. This pathology is observed following prolonged exposure to alcohol [14], and contrast the seemingly beneficial, protective effect of acute but moderate EtOH consumption on cardiac cells and the cardiovascular system at large [14]. To explain this discrepancy the dose of alcohol consumed and the frequency of consumption have been implicated. Because cardiac cells lack cytoplasmic alcohol dehydrogenase (EC 1.1.1.1), which rapidly oxidize doses of ethanol smaller than 35–40 mM (≤ 0.25% v/v) attention has focused on the alcohol-inducible cytochrome P450-2E1 (cyP4502E1, EC 1.14.13.n7) located within the cardiac sarcoplasmic reticulum [16]. CyP4502E1, like alcohol dehydrogenase, catalyzes the oxidation of EtOH to acetaldehyde, the moiety to which numerous deleterious effects within the cardiac cell have been attributed [17]. In addition, the reaction catalyzed by the cyP4502E1produces also reactive oxygen species (ROS) and free radicals, which further alter cardiac cell functions and bioenergetics, contributing to the development of alcoholic cardio-myopathy.

The present study investigated the effects of short- and long-term effect of EtOH exposure in H9C2 cells, a widely used *in-vitro* model of cardiac myocytes. The reported results indicate that a dose- and time-dependent magnesium extrusion from these cells, which affects all the major cellular compartments we could reliably assess. The effect of EtOH is reduced by inhibitors of cyP4502E1 metabolism and ROS formation, suggesting that these two mechanisms are essential to mobilize Mg^2+^ from the cells. Taken together, these data indicate that loss of cellular Mg^2+^ is an integral component of the effect of EtOH on cardiac cells. The Mg^2+^ loss affects all main cellular organelles (i.e. cytoplasm, mitochondria, and sarcoplasmic reticulum, with major implications for ATP production and utilization, and Ca^2+^-dependent cardiac contractility within the heart.

## Materials and Methods

### Materials

All chemicals were of analytical grade (Sigma, St. Louis). H9C2 cells were from ATCC (Manassas, VA).

### H9C2 cell culture

Culture of H9C2 cells (ATCC) were plated at the concentration of 1 × 10^5^ cells/ml in DMEM medium (Gibco), in the presence of 10% FCS, and maintained in 10% CO_2_ atmosphere. Cells at 80% confluence were used to assess EtOH-induced Mg^2+^ extrusion. The day of the experiment, cells were removed from the incubator, and their media replaced with a Mg^2+^ free medium having the following composition (mM): 120 NaCl, 3 KCl, 1.2 CaCl_2_, 1.2 KH_2_PO_4_, 10 NaHCO_3_, 5 glucose, 10 HEPES, pH 7.2/NaOH, at 37°C [6,7]. Cell plates were placed on a slide warmer set at 37°C, and the cell assessed for Mg^2+^ extrusion by addition of EtOH in the absence or in the presence of 4-methyl-pyrazole (4-MP, 50 M) or chloromethiazole (CMZ, 100 M). Two aliquots of the medium (0.2 ml) were removed at 2 min interval prior to EtOH addition to establish extracellular Mg^2+^ baseline. Following EtOH addition, the incubation was continued for 90 minutes, withdrawing 0.2 ml aliquots of the medium at 15 min intervals. The medium aliquots were sedimented at 7,000 rpm × 1 min in microfuge tubes to exclude possible artifacts due to cell lifting. The supernatant was transferred to clean tubes and assessed for Mg^2+^ content by atomic absorbance spectrophotometry (AAS). At the end of the experiment, the plate was placed on ice, and any residual medium was removed by vacuum aspiration. Cells were rapidly washed (1 ml x 2) with ice-cold 250 mM sucrose, and 0.5 ml 10% HNO_3_ were used to scrape the cells off the well. The cell pellets were digested overnight in 10% HNO_3_. Following sedimentation of the denatured protein (8,000 g for 5 min) in microfuge tubes, Mg^2+^ and Na^+^ contents of the acid extract were measured by AAS in an Agilent 340 properly calibrated.

### Cellular Mg^2+^ distribution

Total cellular Mg^2+^ content and distribution among cytoplasm, mitochondria, and other cellular organelles (e.g. sarcoplasmic reticulum and nucleus) was assessed as reported [18]. Briefly, H9C2 cells were washed, and incubated in Mg^2+^-free medium as described above. Digitonin (50 g/ml final concentration), carbonyl cyanide p-trifluoromethoxyphenylhydrazone (FCCP, 2 g/ml), and A23187 (2 g/ml) were sequentially added to the incubation system at 5 min interval, and aliquots of the incubation medium were withdrawn and sedimented at 5,000 g for 2 min to exclude possible artifacts due to protein or lifted cell. The 5 min interval between agent additions was used because preliminary observation has proven this lapse of time as optimal to mobilize Mg^2+^ from cytoplasm (digitonin), mitochondria (FCCP) and non-mitochondrial pools (A23187). Magnesium content in the supernatants was measured by AAS. Residual Mg^2+^ content in cell pellets was measured by AAS after acid digestion performed as reported previously. The Mg^2+^ content present in the cell pellet and in the extracellular medium prior to the addition of any stimulatory agent were calculated and used as baseline reference to determine the net amount of Mg^2+^ retained within the cell or released into the incubation medium, respectively.

### Oxidative modification of proteins

To determine whether EtOH administration induced Mg^2+^ extrusion via reactive oxygen species formation and oxidative modification of proteins, H9C2 cells were pretreated with 100 M N-acetyl-cysteine (NAC) or 100 M di-thio-threitol (DTT) for 15 min prior to EtOH administration at 37°C. Following EtOH addition, aliquots of supernatant were removed and assessed for Mg^2+^ extrusion by AAS as reported previously. At the end of the experimental procedure, residual medium was removed and the cells were washed twice with fresh medium before being resuspended in lysis buffer and prepared for SDS-PAGE. Western Blot analysis was performed with antibodies recognizing HNE/protein adducts [19] to assess for oxidative modification of proteins. Densitometry was performed using Scion Image Program (NIH). Band density of HNE/protein adducts was normalized to β-actin.

### Additional procedures

Aliquots of the incubation medium were collected at 5 min interval, and LDH activity measured by enzymatic kit (Sigma) sensitive to detect changes in the U/ml range, and expressed as U/L. LDH activity was assessed as a percentage of the total amount of the enzyme releasable from digitonin-permeabilized cells.

Protein content was determined by Lowry assay [20] using bovine serum albumin as a standard.

### Statistical analysis

The data are reported as mean ± SE. Data were first analyzed by one-way ANOVA. Multiple means were then compared by Tukey’s multiple comparison test performed with a q-value established for statistical significance of P<0.05.

## Results

H9C2 cells were used as an in-vitro model to investigate the effect of short- and long-term exposure to EtOH on cellular Mg^2+^ homeostasis.

**Figure 1:**
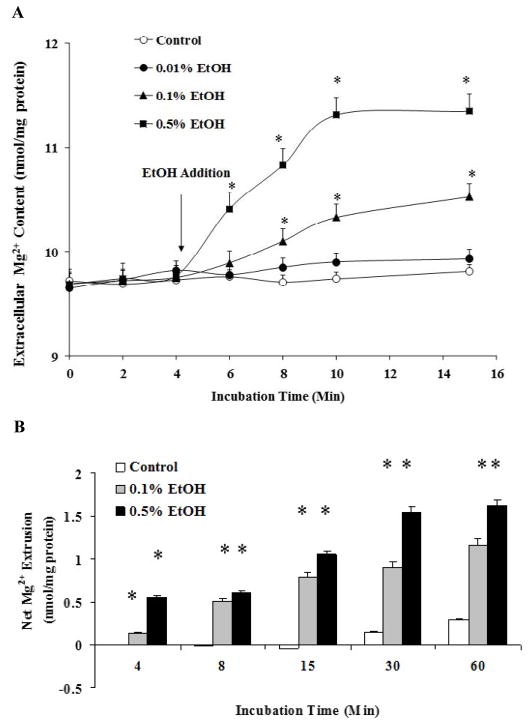
Ethanol-induced Mg^2+^ extrusion in H9C2 cells H9C2 cells, plated as indicated under Material and Methods, were stimulated by addition of varying doses of EtOH to the incubation medium. At the time points reported in the figure, aliquots of extracellular medium were withdrawn and Mg^2+^ content was assessed by AAS. Figure 1A reports a typical Mg^2+^ extrusion profile for H9C2 cells. Net Mg^2+^ extrusion is reported in Figure 1B. Data reported in Figure 1A and 1B are means ± S.E. of 5 different cell preparations, each tested in duplicate for all the experimental conditions. *Statistical significant (p<0.01) vs. corresponding time points in control sample and 0.01% stimulated cells.

**Figure 2:**
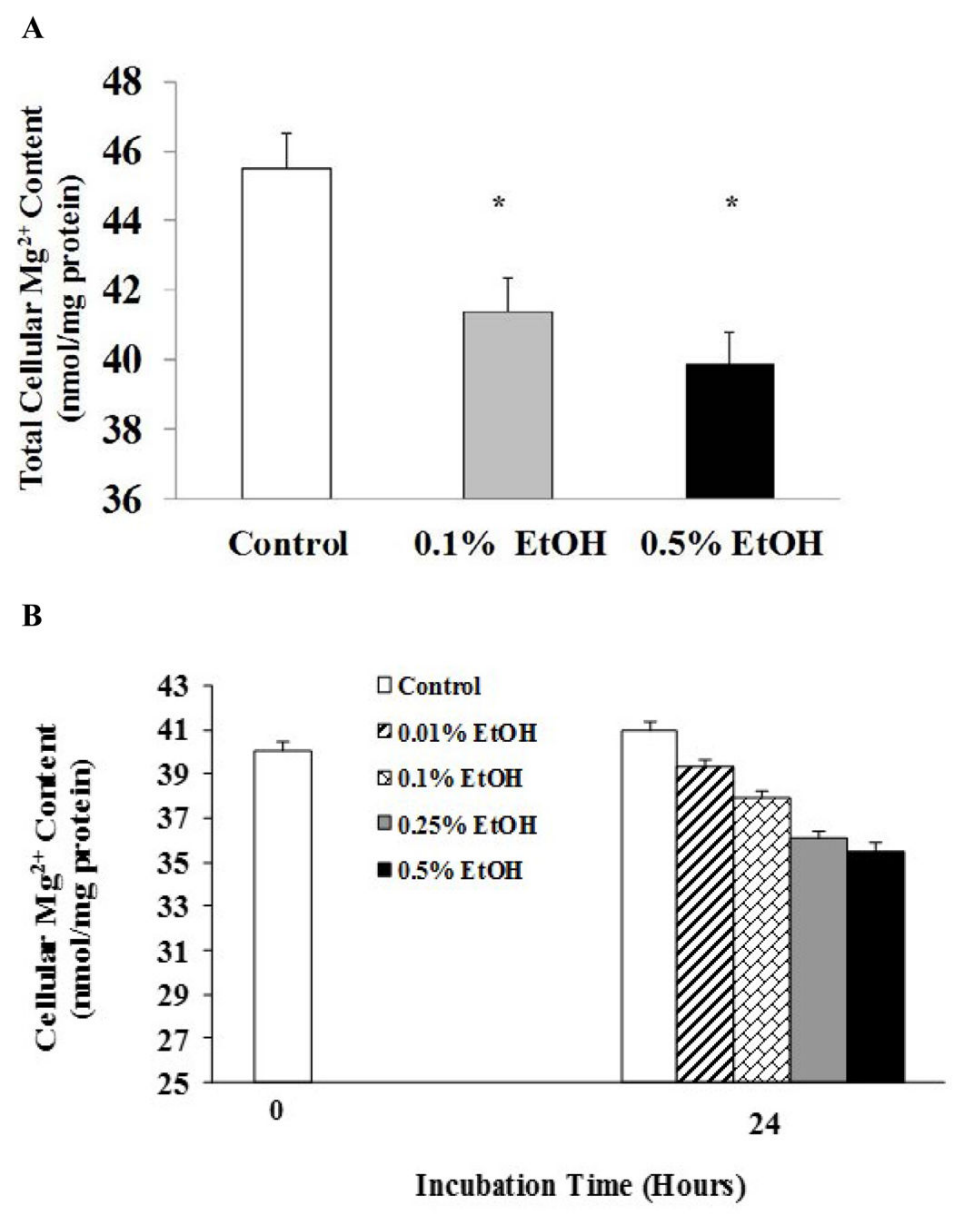
Ethanol-induced Mg^2+^ loss in H9C2 cells H9C2 cells, plated as indicated under Material and Methods, were stimulated by addition of EtOH to the incubation medium. At the end of the experimental protocol, residual medium was removed and cells were digested in 10% HNO_3_. Cellular Mg^2+^ content was assessed by AAS in the acid extract upon sedimentation of the denatured protein as indicated under Materials and Methods. Figure 2A reports residual cellular Mg^2+^ content following 60 min stimulation with the reported doses of EtOH. Figure 2B reports residual cellular Mg^2+^ content following 24h stimulation with the reported doses of EtOH. Data reported in Figure 2A and 2B are means ± S.E. of 4 different cell preparations, each tested in duplicate for all the experimental conditions. *Statistical significant (p <0.01) vs. corresponding time points in control sample.

As Figure 1 shows, H9C2 cells exposed to EtOH extruded Mg^2+^ across the cell membrane into the extracellular medium in a dose- and a time-dependent manner (Figure 1A). The Mg^2+^ extrusion was observed as net increase in the extracellular medium (Figure 1B), or as a decrease in total cellular Mg^2+^ content (Figure 2A). Exposure to EtOH for 24 h also resulted in a detectable Mg^2+^ loss (Figure 2B) that was slightly higher than that observed in cells exposed to EtOH for 60 min (Figure 2A).

**Figure 3:**
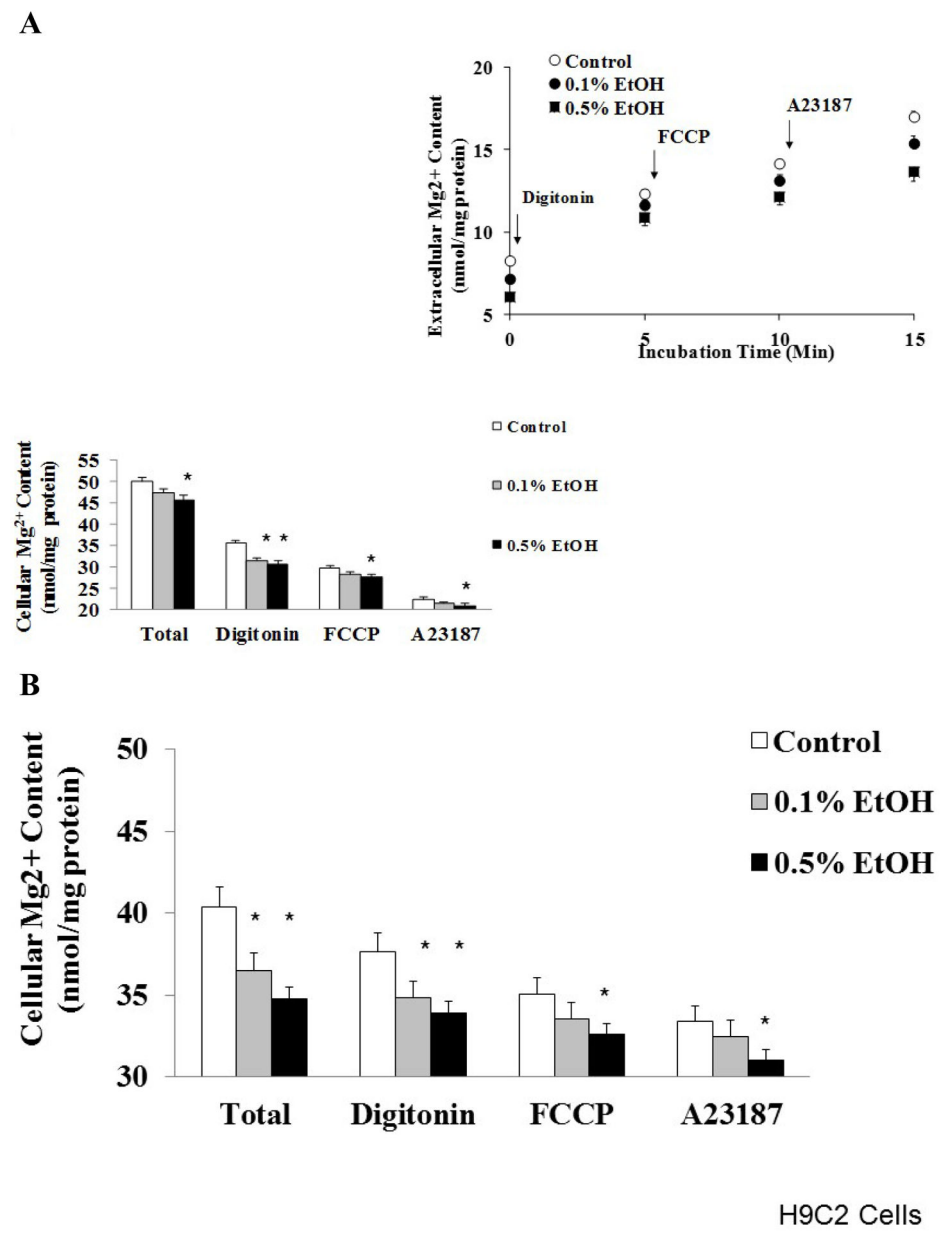
Cellular Mg^2+^ distribution following EtOH stimulation H9C2 cells were harvested and stimulated in vitro with different doses of EtOH. Following 60 min stimulation (Figure 3A), cells were sedimented (800 g x 2 min) and gently resuspended in Mg^2+^ free-medium (see Materials and Methods). Following a few minutes of equilibration, digitonin, mitochondrial uncoupler (FCCP) and ionophore (A23187) were added sequentially to mobilize Mg^2+^ from different cellular compartments (Figure 3A, inset). Estimated Mg^2+^ contents present in cytoplasm, mitochondria, and non-mitochondrial compartments are reported in Figure 3A. For the experiment reported in Figure 3B, H9C2 cells were stimulated in vitro for 24 h with the reported doses of EtOH before being harvested and treated as reported above. Data reported in Figure 3A (including onset) and Figure 3B are means ± S.E. of 4 different cell preparations, each tested in duplicate for all the experimental conditions. *Statistical significant (p<0.01) vs. corresponding time point in control sample.

To determine whether EtOH-induced Mg^2+^ extrusion depleted specific cellular compartments, H9C2 cells were sequentially treated with digitonin, FCCP, and A23187 following 60 min or 24 hours exposure to different doses of EtOH. We have successfully used this approach to quantitate Mg^2+^ from the cytoplasm (digitonin), mitochondria (FCCP) and other, non-mitochondrial, cellular pools (A23187) in other cell models [18,21]. The results reported in Figure 3A indicate that EtOH administration depleted all cellular Mg^2+^ pools in a dose-dependent manner. A similar ubiquitous depletion was observed in H9C2 cells treated with 0.5% EtOH for 24 hours (Figure 3B). The loss of Mg^2+^ induced by EtOH was associated with a decrease in total ATP content: 4.32 ± 0.044 nmol/mg protein in control cells vs. 3.98 ± 0.037 nmol/mg protein in 0.1% EtOH treated cells vs. 3.76 ± 0.042 nmol/mg protein in 0.2% EtOH treated cells vs.3.52 ± 0.041 nmol/mg protein in 0.5% EtOH-treated cells. At the two lower EtOH concentrations the decrease in cellular ATP did not achieve statistical significance but the downward trend persisted throughout all the EtOH concentrations tested. For the 0.5% EtOH dose the decrease in phosphonucleotide content was approx. 17% than the corresponding Mg^2+^ loss (approximately 25%, Figure 3B).

**Figure 4:**
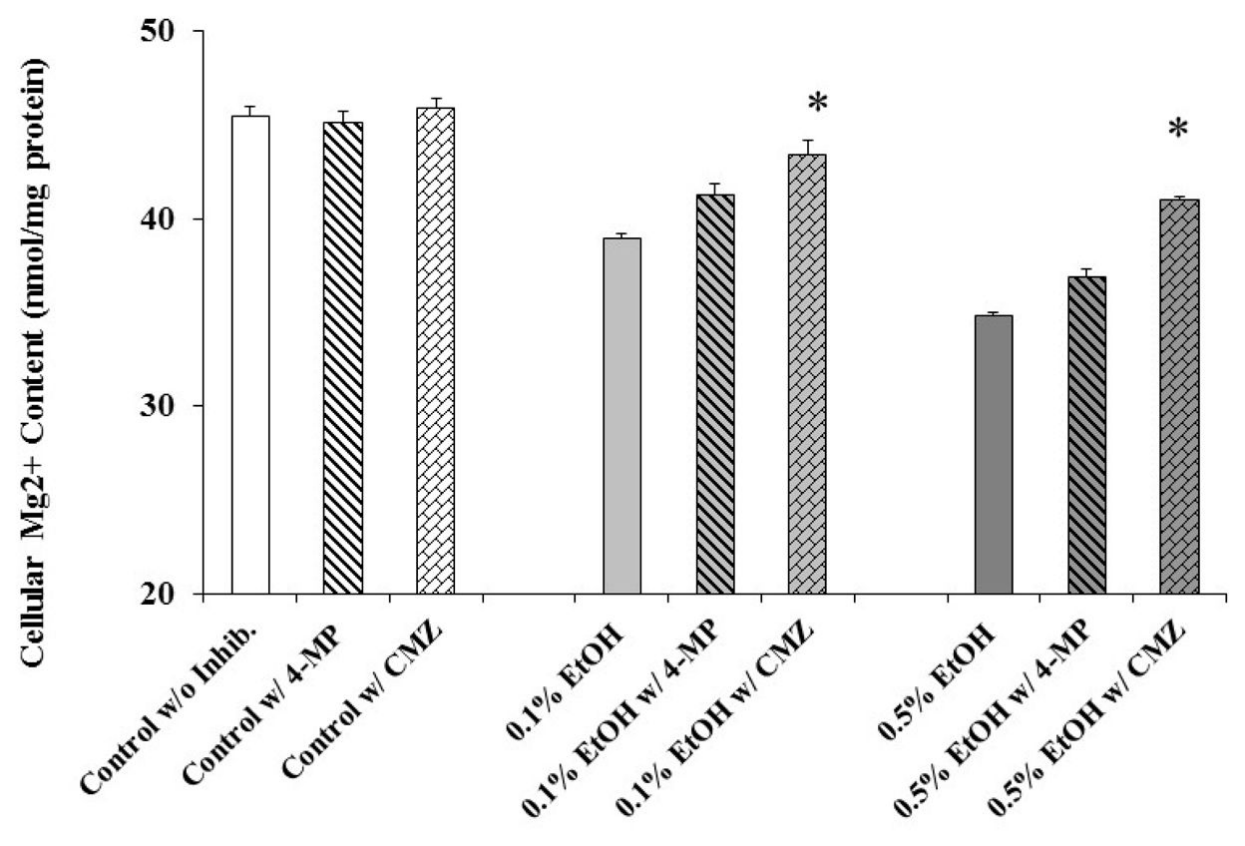
Inhibitory effect of CMZ and 4-MP on EtOH -induced Mg^2+^ extrusion in H9C2 cells H9C2 cells, plated as indicated under Material and Methods, were stimulated by addition of 0.1% or 0.5% EtOH to the incubation medium in the presence of chloromethiazole (CMZ, 100 M) or 4-methyl-pyrazole (4-MP, 50 M). At the time points reported in the figure, aliquots of extracellular medium were withdrawn and Mg^2+^ contents assessed by AAS. For simplicity, net Mg^2+^ extrusion at t=60 min is reported. Data are means ± S.E. of 5 different cell preparations, each tested in duplicate for all the experimental conditions. *Statistical significant (p<0.01) vs. corresponding time point in EtOH-treated sample in the absence of inhibitors.

In cardiac cells, EtOH is oxidized to acetaldehyde by the reticular cytochrome P450-2E1 (CYP2E1, EC 1.14.13.n7), in a reaction coupled with the production of reactive oxygen species, which 0 in turn – lead to free radicals and lipid peroxidation generation within the cell [17]. In liver cells, inhibition of alcohol metabolism prevents ATP loss and Mg^2+^ extrusion in liver cells [11]. Hence, we assessed the ability of chloromethiazole (CMZ), a specific inhibitor of CYP2E1 to prevent Mg^2+^ extrusion by blocking ethanol metabolism [22]. For comparison, we used 4-methyl-pyrazole (4-MP). This compound is more effective at inhibiting the alcohol dehydrogenase and inhibits the cyP4502E1 only partially [22]. As Figure 4 shows, administration of CMZ resulted in a larger retention of cellular Mg^2+^ within the cell following 60 min exposure to 0.1% or 0.5% EtOH while 4-MP pre-treatment was much less effective. Administration of 100 M CMZ inhibited the effect of 0.1% and 0.5% EtOH at all the time points (not shown) and reduced net Mg^2+^ extrusion at t = 60 min by more than 50% (Figure 4). Administration of 150M CMZ inhibited Mg^2+^ extrusion by approximately 70% (not shown). No higher concentrations of CMZ were tested. In contrast, administration of 50 M 4-MP to H9C2 cells inhibited 0.1% EtOH-induced Mg^2+^ extrusion by <25% at all time points (not shown) and reduced net Mg^2+^ extrusion at t=60 min by ~30% (Figure 4), consistent with the partial inhibition of CYP2E1 activity by this agent [22]. When H9C2 cells were challenged with 0.5% EtOH, the protection provided by 4-MP was ~30% at all time points (not shown) including t=60 min (Figure 4). Administration of 100 M 4-MP did not provide a more effective protection (not shown), and higher concentrations of 4-MP were not tested. Co-addition of 4-MP (50 M) and CMZ (100 M) did not attain higher inhibition than the one observed with CMZ alone (not shown).

**Figure 5:**
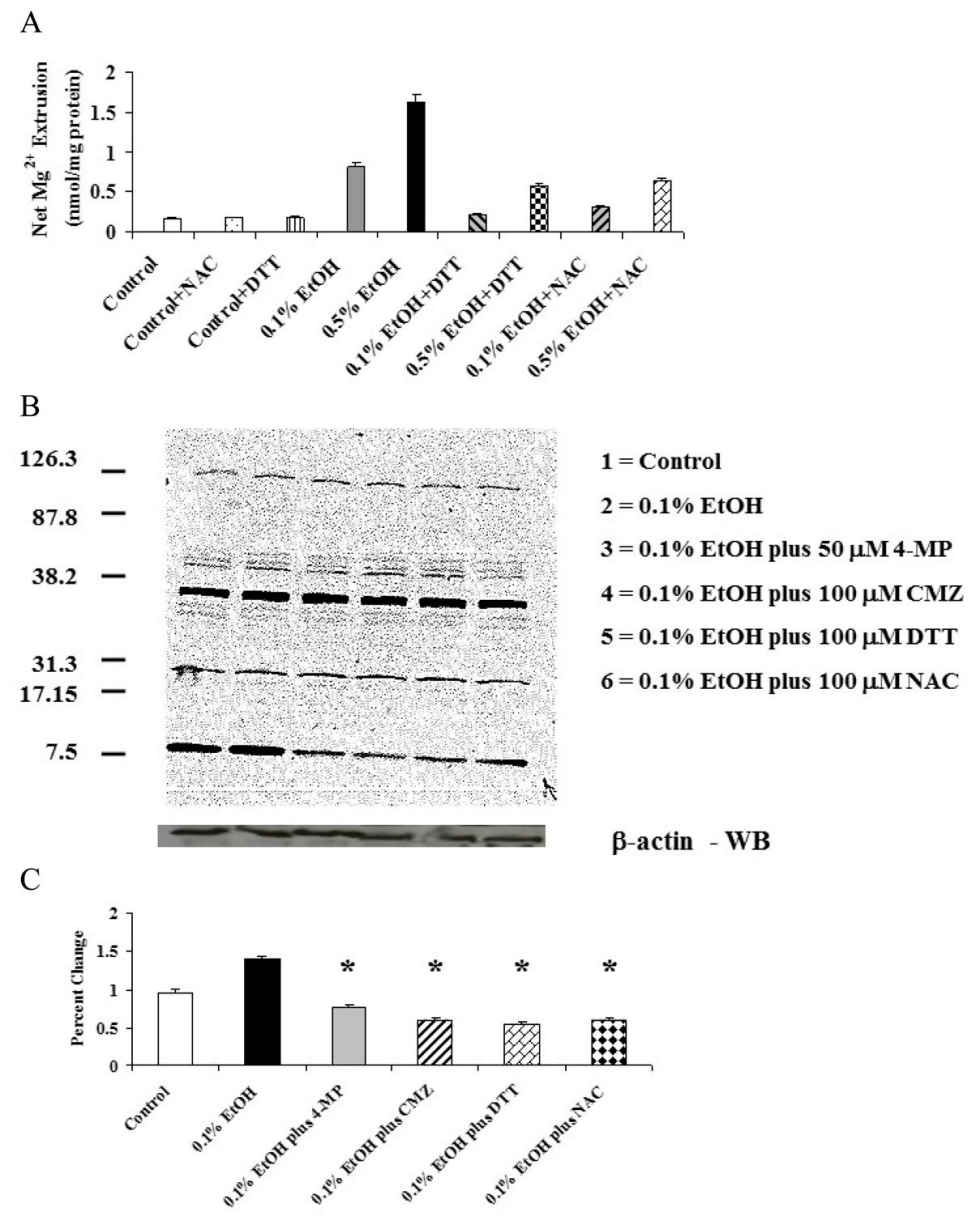
Inhibitory effect of DTT and NAC on EtOH-induced Mg^2+^ extrusion in H9C2 cells H9C2 cells, plated as indicated under Material and Methods, were stimulated by addition of 0.1% or 0.5% EtOH to the incubation medium in the presence of dithio-threitol (DTT, 100 M) or N-acetyl-cysteine (NAC, 50 M). At the time points reported in the figure, aliquots of extracellular medium were withdrawn and Mg^2+^ contents assessed by AAS. Figure 5A reports net Mg^2+^ extrusion at t = 60 min for simplicity. Figure 5B reports typical western Blot experiments for the formation of HNE-protein adducts in H9C2 cells treated with EtOH in the absence and in the presence of DTT or NAC. A β-actin western blot is reported for loading comparison purposes. Densitometry of 4 different similar experiments is reported in Figure 5C. Data in Figure 5A and 5C are means ± S.E. of 4 different cell preparations. For the data in Figure 5A, each cell preparation was tested in duplicate for all the experimental conditions. *Statistical significant (p<0.01) vs. corresponding time point in EtOH-treated sample in the absence of inhibitors.

To assess the involvement of reactive oxygen species and acetaldehyde formation in EtOH-induced Mg^2+^ extrusion, H9C2 cells were pre-treated with N-acetyl-cysteine (NAC) or di-thio-threitol (DTT) for 15 min prior to EtOH administration. The results reported in Figure 5A indicate that both DTT and NAC prevented EtOH-induced Mg^2+^ extrusion to a significant extent (60% to 65%) irrespective of the dose of EtOH administered. Consistent with this protective effect, Western blot analysis indicated a decrease in HNE modified cardiac proteins (Figures 5B and 5C), suggesting a reduced formation of ROS and lipid peroxidation products.

## Discussion

Consumption of small doses of EtOH has beneficial effects on the cardiovascular system especially if alcohol consumption is intermittent [14]. In contrast, prolonged consumption of alcohol, especially in high doses, results in the development of alcoholic cardiomyopathy in human subjects [14,15]. The disease has been attributed to the oxidation of EtOH to acetaldehyde by the cytP4502E1 located within the sarcoplasmic reticulum of the cardiac myocyte [16], and the coupled production of reactive oxygen species, free radicals, and lipid peroxidation products, which all react readily with phospholipids, signaling proteins, and enzymes [16]. As cytP450-2E1 activity is induced by high doses of EtOH, its enzymatic activity can reasonably explain the different effects of moderate *versus* chronic EtOH consumption on cardiac functions. The production of large quantities of acetaldehyde depresses the cardiac contractile function [23] and results in the release of significant amounts of troponin C into the extracellular space [24]. Combined, these processes affect cardiac contractility and cardiac ejection fraction to a significant extent, setting the conditions for the development and progression of alcoholic cardiomyopathy.

While attention has been paid to the effect of acute and chronic EtOH administration on cellular and reticular Ca^2+^ homeostasis and its impact on myocyte contractility, no information is currently available as to whether EtOH metabolism affects cardiac Mg^2+^ homeostasis. Magnesium is abundantly present with the cardiac myocyte, and evidence in the literature indicates that cellular and extracellular Mg^2+^ play major roles in cardiac physiology [1–3] by controlling action potential duration and regulation of Na^+^ and Ca^2+^ channels [25]. Conversely, an increased risk of ischemic heart disease [26] and specific forms of arrhythmias including the long QT syndrome have been associated with a less than optimal Mg^2+^ content within the cardiac tissue [27].

More specifically for the chronic ethanol administration, Mg^2+^ supplementation has been reported to ameliorate the myocardial dysfunction associated with alcoholic cardiomyopathy, renormalizing heart size, isometric force and isotonic shortening [15]. How exactly Mg^2+^ elicits these effects has not been investigated. Because Mg^2+^ acts as a natural Ca^2+^-channel blocker, it is possible that cardiac force development and cardiac cell shortening depend on the restoration of normal cytosolic Ca^2+^ levels, especially in diastoles, when the effect of abnormally elevated resting Ca^2+^ levels directly impact on contractile myofilaments’ function. Less clear is whether restoring physiological Ca^2+^ levels within the cardiac myocyte attenuates Ca^2+^-mediated signaling leading to hypertrophy [28].

The present study investigated the effect of EtOH administration on Mg^2+^ homeostasis in an in-vitro model of cardiac myocytes. The results suggest that EtOH metabolism via cyP4502E1 promotes Mg^2+^ loss from cytoplasm, sarcoplasmic reticulum, and mitochondria (albeit to a lesser extent). Evidence for the occurrence of such a loss is that: 1) total cellular and subcellular Mg^2+^ decrease in a time-dependent manner that directly correlates to the dose of EtOH administered, and 2) inhibition of EtOH metabolism prevents the Mg^2+^ loss from cardiac cells. Evidence that Mg^2+^ loss from cardiac cells depends on alcohol oxidation and related processes is provided by the protective effect of CMZ, an inhibitor of cytP4502E1 activity, DTT and NAC, two anti-oxidant agents.

Cytoplasm, mitochondria, and sarcoplasmic reticulum are the three main Mg^2+^ compartments within nucleated mammalian cells including cardiac myocytes [1–3], and Mg^2+^ plays a significant role in each of them by controlling ATP production, cardiac bioenergetics, and Ca^2+^ release and cycling, respectively [1–3]. Thus, loss of Mg^2+^ from these compartments can affect cellular bioenergetics and metabolic processes to a varying extent. Adenosine triphosphate is the main agent forming a complex with Mg^2+^ within the cytoplasm and the mitochondrial matrix. Hence, loss of ATP as a result of EtOH administration [11] limits the ability of the cell to retain Mg^2+^ in cytoplasm and mitochondria. In turn, mitochondria depend on proper Mg^2+^ homeostasis and Ca^2+^/Mg^2+^ ratio for proper dehydrogenases activity and to maintain an optimal ATP level for the cardiac myocyte [29]. A decrease in matrix Mg^2+^ level has been associated with structural and functional alteration of mitochondrial complexes and dehydrogenases [29] and with a marked decrease in mitochondrial respiratory rate [29]. The decreased utilization of oxygen by the mitochondrial electron chain has been related to the increased production of reactive oxygen species (ROS) [30]. This mitochondrial mechanism would be in addition to ROS production as a byproduct of cytP4502E1 activity. Hence, the increase in ROS production and associated lipid peroxidation observed following EtOH administration can be largely explained through these mechanisms. In turn, loss of Mg^2+^ from the various cellular organelles can depend on changes in membrane integrity following EtOH metabolism-related ROS and free radicals formation and associated lipid peroxidation.

It could be argued that the loss of Mg^2+^ observed in this in vitro model (i.e. about 2 nmol Mg^2+^/mg protein over 60 min, or ~5 nmol Mg^2+^/mg protein over 24h) is too small to be significant in absolute terms and of relevance for the development of alcohol related cardiomyopathy. It has to be considered, however, that when re-calculated for the cell volume, a loss of 5–6 nmol Mg^2+^/mg protein actually represents a loss equivalent to approximately 10% of the total cellular Mg^2+^ content. The average rat cardiac myocytes volume is estimated at ~30–35 pL [31] while initial estimates for the cardiac myocytes cell line used in our study suggest a cell volume of ~25 pl (Romani, unpublished observation). When taking into account these average cell volumes the Mg^2+^ losses observed under our experimental conditions accounts for a ~1.5–2 mM decrease in total Mg^2+^ content (out of a total cardiac myocytes concentration of ~16–20 mM [1,6,8,10]). When reported to the volume of organelles such as virtual cytoplasm (i.e. cytoplasm devoid of contractile filaments), mitochondria and sarcoplasmic reticulum, in which they were observed to occur, these losses can reflect higher decreases in Mg^2+^ concentration, and have a much stronger impact on the physiological operation of specific enzymes or proteins located within these compartments.

To our knowledge, this study is the first to investigate EtOH-induced changes in Mg^2+^ homeostasis in an in-vitro model of cardiac cells largely used to study cardiac hypertrophy. The results of this study unveils interesting new lines of research to elucidate the mechanisms responsible for Mg^2+^ loss in cardiac cells, and the potential relevance for cell bioenergetics, contractile function, and cardiac hypertrophy.

## Abbreviations

4-MP: 4-Methyl-Pyrazole
cyP4502E1: Cytochrome P450-2E1
CMZ: Chloromethiazole
EtOH: Ethanol
DTT: Dithiothreitol
FCCP: Carbonyl Cyanide p-trifluoromethoxyphenylhydrazone
[Mg^2+^] i: Cytoplasmic Free Magnesium Concentration
NAC: N-Acetyl-Cysteine
